# Mycophenolate mofetil as second line treatment in autoimmune hepatitis – A retrospective single center analysis

**DOI:** 10.1016/j.jtauto.2022.100172

**Published:** 2022-11-19

**Authors:** Mirjam Kolev, Stefan Diem, Lara Diem, Susana G. Rodrigues, Annalisa Berzigotti, Guido Stirnimann, Nasser Semmo

**Affiliations:** a Department of Visceral Surgery and Medicine, Inselspital, Bern University Hospital, University of Bern, Switzerland; b Department of Neurology, Inselspital, Bern University Hospital, University of Bern, Switzerland

**Keywords:** Autoimmune hepatitis, Autoimmune liver disease, Mycophenolate mofetil, Azathioprine, Second line treatment, AIH, autoimmune hepatitis, ALT, alanine aminotransferase, ANA, anti-nuclear-antibodies, AZA, azathioprine, BDN, budesonide, CI, calcineurin inhibitor, CyA, cylcosporine A, IgG, immunoglobuline G, INR, international normalized ratio, IQR, interquartile range, LKM-1, liver-kidney-microsomal antibodies, MMF, mycophenolate mofetil, PBC, primary biliary cholangitis, PDN, prednisolone, PSC, primary sclerosing cholangitis, SLA, soluble liver antigen antibodies, SMA, smooth-muscle cell antibodies, TNFi, tumor necrosis factor inhibitor, UDCA, ursodeoxycholic acid, ULN, upper limit of normal

## Abstract

**Background:**

Most patients with autoimmune hepatitis respond to standard treatment with steroids and azathioprine. While the disease is usually fatal if untreated, patients who respond well to therapy have an excellent prognosis. Nevertheless, second-line treatment is necessary in approximately 20% of patients, due to either intolerance or insufficient response to first line treatment.

While data for mycophenolate mofetil (MMF) in patients intolerant to azathioprine is encouraging, MMF seems of less benefit in patients with insufficient response to first line treatment, but analyzed data on this issue is limited.

**Aim:**

To evaluate the efficacy and safety of MMF as a second-line therapy in patients with AIH.

**Methods:**

Retrospective analysis of a monocentric database of AIH patients who received medical care from 2000 to 2022. Clinical, immunological and biochemical parameters were assessed at different time points including last follow-up.

**Results:**

Overall, 144 patients with AIH were identified. Fifty out of 144 (35%) AIH patients received MMF. Forty (80%) received MMF due to first line treatment intolerance, while ten (20%) due to insufficient response to first line treatment.

Remission with MMF monotherapy was 81.5% in the intolerance group versus 30% in the insufficient response group. Patients switched to MMF because of an insufficient response, more often needed additional prednisolone doses higher than 5 mg/day, a switch to third-line treatment or combination regiments, to achieve disease control.

**Conclusions:**

Patients treated with MMF because of intolerance to first line treatment show a good disease control under MMF in the majority of cases. Efficacy is considerably lower in the patients switched to MMF because of an insufficient response to first line treatment.

## 1 Introduction

Autoimmune hepatitis (AIH) is an immune-mediated inflammatory disease of the liver parenchyma. While untreated disease is usually fatal, patients who respond well to therapy have an excellent prognosis [1]. Liver disease progression can be prevented in most patients who have achieved complete biochemical remission, defined as normal transaminases and normal immunoglobulin G (IgG) levels [2].

Randomized controlled trials published between 1971 and 1974 established the role of prednisolone alone and in combination with azathioprine (AZA), a non-selective purine-analogue, in the treatment of AIH [[3], [4], [5], [6]]. Current treatment is based upon this data and about 80% of the patients achieve remission [7]. However, more than 20% of patients do not respond to or cannot tolerate AZA [8,9]. This is where mycophenolate mofetil (MMF) became of interest, a selective inhibitor of purine-synthesis with a more lymphocyte-specific mode of action than AZA. Initially, most of the data available for MMF came from solid organ transplantation, where MMF was superior to AZA in preventing acute and chronic allograft rejection with a better tolerability [[10], [11], [12]]. Since the first case report for the successful application of MMF in AIH in 1998 [13], several retrospective case series and one prospective study [14] were published (Supplementary Table 1). However, due to the heterogeneous definition of remission, as well as the varying dosing and reporting of steroid use in addition to MMF, comparability of the results is limited. Reported rates of biochemical improvement vary between 25% and 90%. This discrepancy is partly due to the difference in analysis, as only some studies considered the indication for switching treatment. Evidence points towards a better response to MMF in the intolerance group in most reports [[15], [16], [17], [18], [19]], but not in all [20,21].

Despite the lack of prospective studies, out of clinical necessity, MMF has become the most commonly used second-line treatment for AIH in clinical practice in Europe [22] with recent implementation in clinical practice guidelines as the second line treatment of choice in patients intolerant to AZA or 6-mercaptopurine [1]. For patients with insufficient response to first-line treatment, recommendations are weaker, as efficacy seems to be more limited in this patient group [[15], [16], [17], [18], [19]].

The aim of our retrospective analysis was, to describe the efficacy of MMF as a second line treatment for AIH in our cohort, delineated by the indication for the switch being intolerance versus insufficient response.

## 2 Patients and methods

### 2.1 Patients

All adult patients with AIH who received medical care from 2000 to 2022 at the University Hospital of Bern, a tertiary care center in Switzerland, were retrospectively analyzed. Patients were included if the following criteria were fulfilled: 1) Age ≥18 years, 2) diagnosis of AIH based on the simplified criteria of the International AIH group [23], 3) AZA as the first line treatment, 4) MMF as the second line treatment, 5) and follow-up of at least 6 months after starting of MMF.

Other causes of chronic liver disease, including chronic hepatitis B or C, haemochromatosis, 1-antitrypsin deficiency, drug-induced liver disease or Wilson disease, were excluded. Patients with AIH/primary biliary cholangitis or AIH/primary sclerosing cholangitis overlap syndromes were not excluded. A liver biopsy was available from all patients at the time of diagnosis and fibrosis was classified according to the METAVIR scoring system [24].

For remission induction, patients were treated as per local protocol with prednisolone (PDN) 40–60 mg/day, followed by gradual tapering according to response. Treatment with azathioprine (at a dose of 1–2 mg/kg/day) was started usually two weeks after initiation of prednisolone, according to EASL guidelines [25]. If the patients were switched from AZA to 6-mercaptopurine, the dose was halved.

Remission was defined as a normalization of ALT and IgG [2]. Intolerance or insufficient response to AZA was determined by the treating physician in agreement with international guidelines [2,25]. Following discontinuation of AZA, MMF was started at a dose of 2 × 500 mg/d or 2 × 1g/day.

Clinical, biochemical and immunological parameters were assessed at diagnosis, at start of MMF treatment, at 3, 6, 12–24 months as well as at last follow-up after initiation of MMF, respectively. There was missing data at random, as there were not all parameters at all respective time points for all patients available. Most follow-up data was available for the time period of 12–24 months after treatment start with MMF, which is why this time point is reported in more detail. Missing data was considered in all the calculations done and numbers indicated separately in the results section.

### 2.2 Statistical methods

Categorical variables are expressed as numbers and percentages; continuous variables are expressed as median and interquartile range (25IQR-75IQR) or mean with standard deviation as indicated. The Fisher's exact test was used to compare categorical data between the two groups. The Wilcoxon rank-sum test was used to compare quantitative data between two groups. The Kaplan-Meier method was used to plot remission as a function of time, with comparisons made between the two groups by using the log-rank test. All reported p-values are two-tailed, and p < 0.05 indicated statistical significance. Statistical analysis was performed using STATA Version 16.0.

### 2.3 Ethical approval

The study was approved by the Cantonal Ethics Commission of Bern, Switzerland (2020–02136) and conducted according to the Declaration of Helsinki.

## 3 Results

### 3.1 Study flow chart and baseline characteristics

**Fig. 1:**
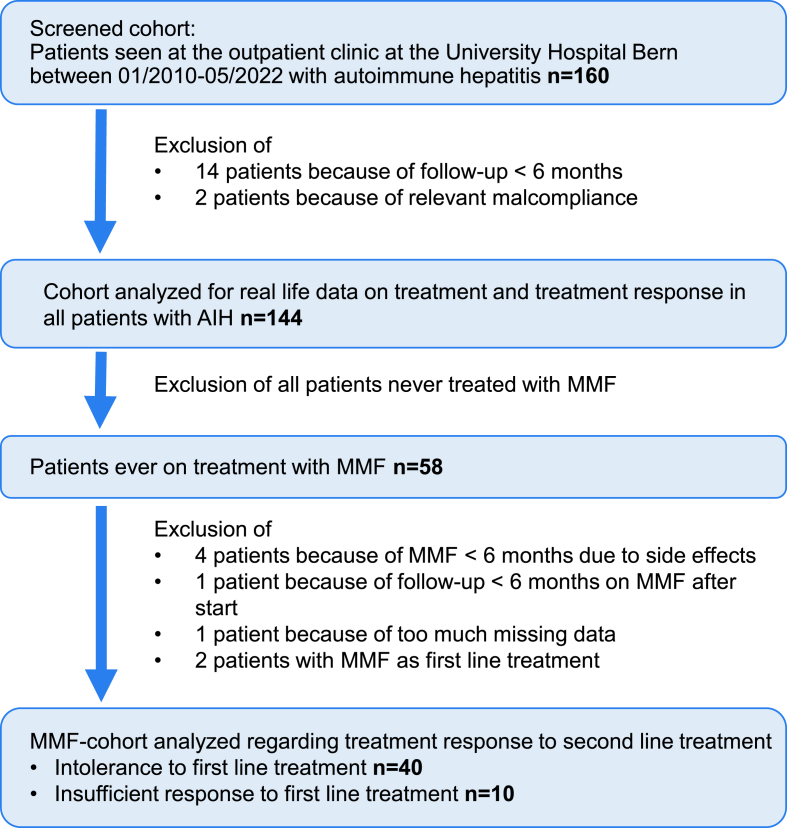
*Study flow chart for patient inclusion.* AIH, autoimmune hepatitis; MMF, mycophenolate mofetil.

The study flow chart in Fig. 1 shows the study population from screening to inclusion for analysis. Overall, 160 patients with AIH were screened and 144 patients met inclusion criteria. Of those, 58 patients (39.5%) received MMF. For the analysis on treatment response to MMF, we identified 50 patients that met inclusion criteria (Fig. 1). Forty (80%) were on second line treatment due to intolerance and ten (20%) due to insufficient response.

The main demographic and laboratory features of the 50 patients treated with MMF are summarized in Table 1. Characteristics were similar in the two groups. Of note, the median time from diagnosis to second line treatment was six (3–19.5) months in the intolerance group versus 49 (9–100) months in the insufficient response group, but did not reach statistical significance (p-value 0.052).

**Table 1:** *Baseline characteristics of all patients on second line treatment with mycophenolate mofetil (MMF).* Categorical variables presented as n (%). Continuous data presented as median and interquartile range (Q25-Q75). If there was missing data for some variables, the number of patients used for the calculations are indicated separately behind the results in the respective cells.

DEMOGRAPHICS	Overall patients on MMF as second line treatment (n = 50)	Delineated by indication for second line treatment	
		Intolerance (n = 40)	Insufficient response (n = 10)
Female	40 (80%)	33 (82.5%)	7 (70%)
Caucasian	43 (86%)	33 (82.5%)	10 (100%)
Age at diagnosis (years)	49.5 (37–58)	49.5 (37.5–57.5)	51 (34–58)
**VALUES AT DIAGNOSIS**			
**Disease activity**			
ALT (<35 U/L in women, <50 U/L in men)	717.5 (350–1175) n = 46	759 (357–1175) n = 37	506 (348–1020) n = 9
Total bilirubin (<17 μmol/l)	31 (16–144) n = 45	44 (15.5–183) n = 36	18 [[16], [17], [18], [19], [20], [21], [22], [23], [24], [25], [26], [27], [28], [29], [30], [31]] n = 9
Immunoglobulin G (<16 g/l)	17.9 (14.3–21.9) n = 37	17.9 (14.3–21.9) n = 29	17.5 (13.5–24.8) n = 8
**Immunserology**			
ANA, Titer ≥1:80	36 (78%) n = 46	31 (82%) n = 38	5 (62%) n = 8
SMA, Titer ≥1:80	32 (71%) n = 45	26 (68.5%) n = 38	6 (86%) n = 7
F-Actin, > 20 Units (measured in SMA positives)	29 (76%) n = 38	23 (66%) n = 35	6 (75%) n = 8
LKM-1, Titer ≥1:80	0 (0%) n = 44	0 (0%) n = 35	0 (0%) n = 9
SLA, > 20 Units	3 (8%) n = 38	2 (6.4%) n = 31	1 (14%) n = 7
**Simplified autoimmune hepatitis (AIH) Score**	7 [[5], [6], [7], [8]] n = 46	7 [[5], [6], [7], [8]] n = 38	6.5 (5–7.5) n = 8
**Assessment of chronic liver disease**			
Fibroscan (kPa)	9.4 (6.7–15.4) n = 22	8.9 (6.3–14) n = 19	21.3 (6.7–30.6) n = 3
Signs of advanced fibrosis/cirrhosis on histology	4 (8%)	2 (5%)	2 (20%)
**VALUES AT START OF MMF**			
Duration of disease from diagnosis to start of MMF	8.5 [[3], [4], [5], [6], [7], [8], [9], [10], [11], [12], [13], [14], [15], [16], [17], [18], [19], [20], [21], [22], [23], [24]]	6 (3–19.5)	49 (9–100)
ALT (<35 U/L in women, <50 U/L in men)	60 (26–105.5) n = 48	47 (23–102) n = 38	85 (64–158)
Total bilirubin (<17 μmol/l)	13 [[8], [9], [10], [11], [12], [13], [14], [15], [16], [17], [18], [19], [20], [21], [22], [23]] n = 43	12 [[7], [8], [9], [10], [11], [12], [13], [14], [15], [16], [17], [18], [19], [20]] n = 33	18.5 [[12], [13], [14], [15], [16], [17], [18], [19], [20], [21], [22], [23], [24], [25]]
Immunoglobulin G (<16 g/l)	11.7 (9.95–13.5) n = 25	11.7 (9.2–13.3) n = 19	12.9 (10.2–18.5) n = 6
**PRESENCE OF OTHER AUTOIMMUNE DISEASES**			
Primary biliary cholangitis	8 (16%)	6 (15%)	2 (20%)
Primary sclerosing cholangitis	1 (2%)	1 (2.5%)	0 (0%)
Extrahepatic autoimmune diseases	28 (56%)	24 (60%)	4 (40%)

ALT, alanine aminotransferase; ANA, anti-nuclear-antibodies; SMA, smooth-muscle cell antibodies; AIH, autoimmune hepatitis; INR, international normalized ratio; LKM-1, liver-kidney-microsomal antibodies; SLA, soluble liver antigen antibodies.

Dosage of MMF was 2 × 1 g in most patients. One patient had 2.5 g/day with a good tolerance. In patients with disease remission, MMF maintenance dose was reduced to 2 × 750 mg/d in two patients, 2 × 500 mg/d in 7 patients and to 2 × 250 mg/d in one patient, respectively.

Before treatment with MMF, 49 (98%) patients had received prednisolone, 50 (100%) AZA and ten (20%) budesonide. One patient (2%) was treated with cyclosporine A (CyA), when MMF was added.

More background information on the overall cohort of patients with AIH treated at our center is provided in Supplementary Figs. 1–3. It includes treatment tree decisions (Supplementary Fig. 1), additional prednisolone use (Supplementary Fig. 2) and remission at last follow-up (Supplementary Fig. 3).

### 3.2 Follow-up 12–24 months after treatment start with MMF

**Fig. 2:**
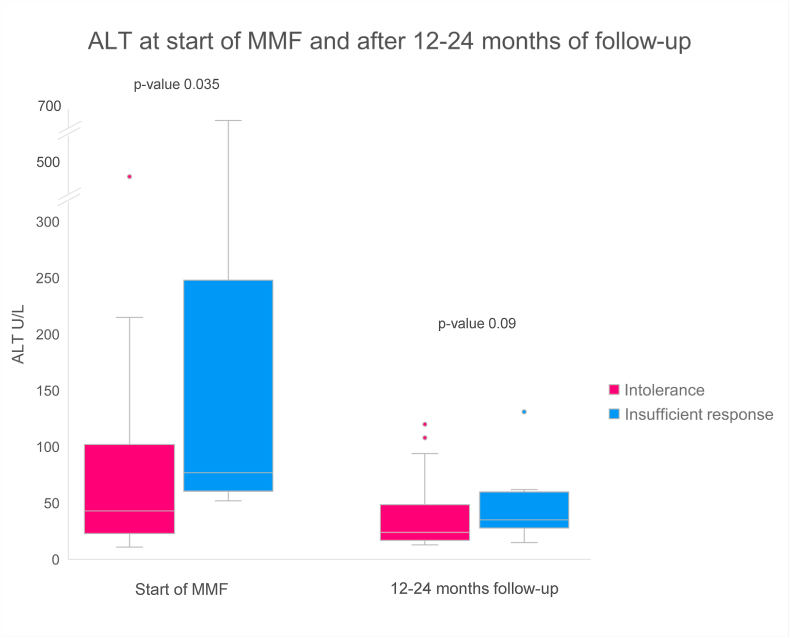
*Median ALT values at start of MMF and after 12–24 months offollow-up.* Data shown as median (exclusive median) with interquartile range. N = 39 for intolerance start MMF, n = 9 for insufficient response start MMF, n = 30 for intolerance follow-up, n = 9 for insufficient response follow-up. MMF, mycophenolate mofetil.

**Fig. 3:**
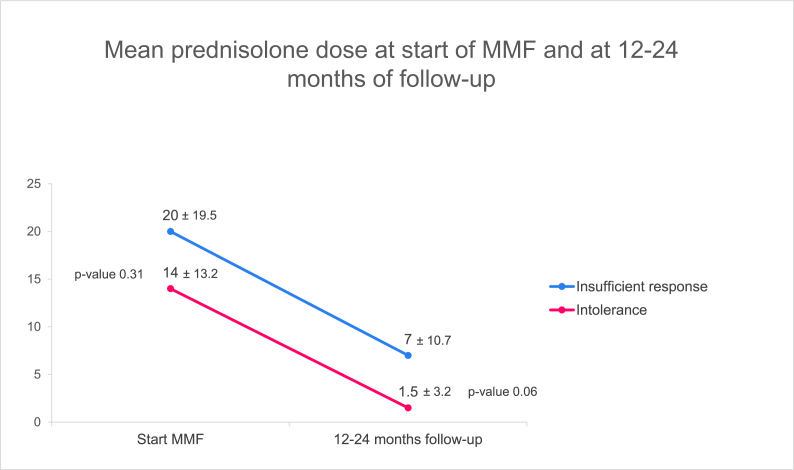
*Mean (SD) dose of prednisolone (mg) at start of MMF and after 12–24 monthsof follow up shown in the two groups.* N = 39 for intolerance start MMF, n = 9 for insufficient response start MMF n = 30 for intolerance follow-up, n = 9 for insufficient response follow-up. MMF, mycophenolate mofetil; SD, standard deviation.

In Fig. 2 median ALT at start of MMF treatment and after 12–24 months are shown and not significantly different. In Fig. 3 additional prednisolone treatment at these time points are shown, without statistically significant differences.

#### 3.2.1 Intolerance group

At the time point 12–24 months of treatment start, three (7.5%) out of 40 patients in the intolerance group had stopped MMF, two at the patient's own request and stable disease, and one for the desire to father a child. For another six patients data was not available. Of the 31 patients analyzed, 23 (74%) had a normal ALT, three (10%) patients an ALT <2xULN and five (16%) patients an ALT >2xULN. IgG was available in 21 patients and of those, it was normal in 18 patients (86%), <1.1xULN in one (4.5%) patient and >1.1xULN in two (9.5%) patients. In the 21 patients where both values were available, 14 (67%) were in remission with normal ALT and IgG, six (28.5%) patients had one of the values elevated and one (4.5%) patient had both values elevated. One patient with elevated IgG had cirrhosis.

#### 3.2.2 Insufficient response group

All ten patients in the insufficient response group were still under treatment with MMF and of nine, 12–24 months follow-up data were available. Of the nine patients, five (56%) had a normal ALT, three (33%) patients <2xULN and one (11%) patient an ALT >2xULN. IgG values were only available in four patients and were normal in all. However, of these four patients all but one also had normal ALT levels.

### 3.3 Efficacy of MMF to control disease activity

**Fig. 4:**
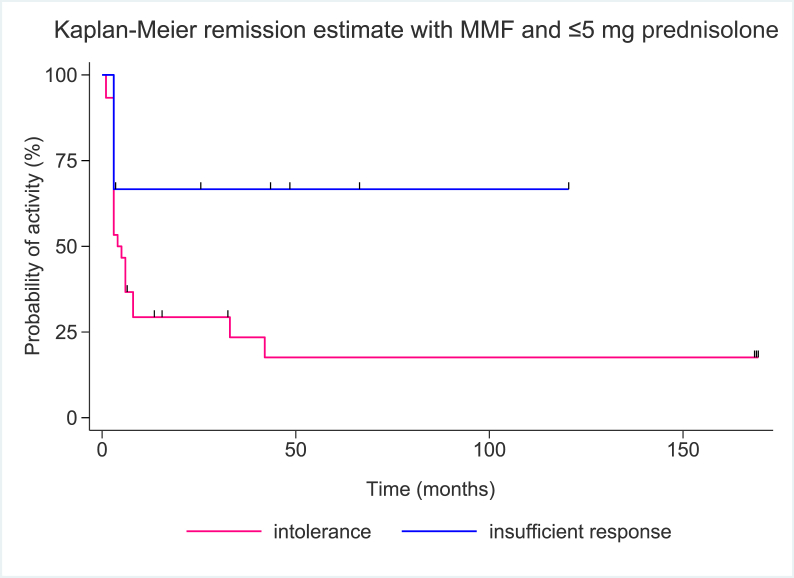
*Kaplan-Meier estimate of remission on MMF and up to ≤*5 mg *PDN.* Censored patients are indicated.

The intolerance group had a higher probability of disease control in comparison to the insufficient response group. This is reported in a Kaplan-Meier analysis on remission on MMF and up to 5 mg PDN delineated by the two groups in Fig. 4.

**Fig. 5:**
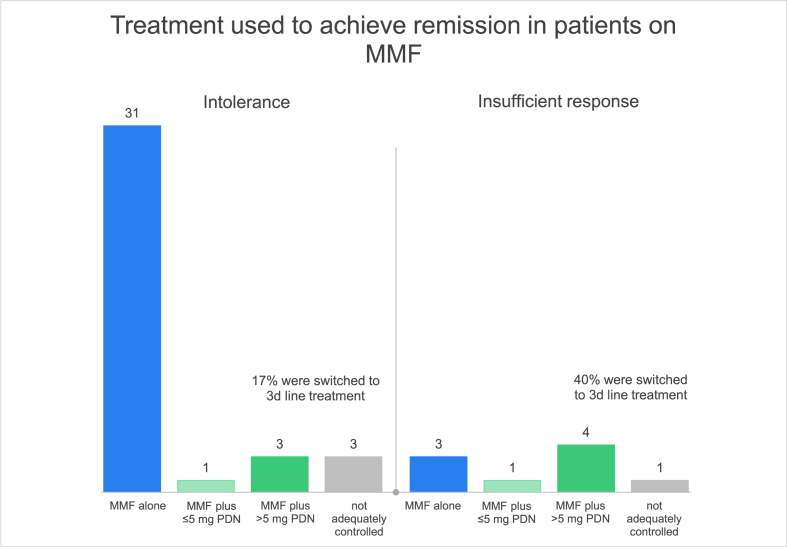
*Efficacy of MMF delineated by the two groups.* If patients were not in remission with these combinations, we stated it as ‘not adequately controlled with MMF’. Three patients did not match any of the groups, which is why they are not represented in Fig. 5 n = 38 for the intolerance group and n = 10 for the insufficient response group. 31 (81%) patients in the intolerance group achieved remission with monotherapy versus three (30%) patients in the insufficient response group. MMF, mycophenolate mofetil; PDN, prednisone.

Furthermore, we grouped patients according to remission with monotherapy or two different levels of additional prednisolone doses (Fig. 5).

Four patients are not represented in Fig. 5, because they did not match any of the groups, either because of reduced MMF doses or no follow-up after treatment adjustment.

Disease remission rate on monotherapy with MMF for the intolerance was higher than in the insufficient response group (P = 0.008). This was also observed in patients on MMF plus ≤5 mg PDN (P = 0.023).

### 3.4 Treatment at last follow-up

#### 3.4.1 Intolerance group

Of the 40 patients treated with MMF because of intolerance to AZA, 25 (62.5%) patients were still on MMF treatment at last follow-up. Nine (22.5%) patients had stopped treatment because of stable remission. Overall, three (7.5%) patients were switched to third line treatment due to intolerance to MMF and three (7.5%) were switched to third line treatment because of insufficient response to MMF. One (2.5%) patient was switched to third-line therapy with CyA for the desire to father a child.

#### 3.4.2 Insufficient response group

Of the 10 patients treated with MMF due to insufficient response, all (100%) patients were still on immunosuppression at last follow-up. Five (50%) patients were on MMF at last follow-up. Two as a monotherapy, one with additional 2.5 mg and 7.5 mg PDN, each, and one with CyA and 5 mg PDN.

Of those five patients not on MMF anymore at last follow-up, one patient (10%) was switched to third-line treatment due to intolerance. Two (20%) patients were switched to third-line treatment because of insufficient response to MMF. In one (10%) patient MMF was stopped because rituximab was introduced to treat a cerebral lymphoma. In one patient MMF was changed back to AZA and PDN during the period when she wished to become pregnant.

### 3.5 Follow-up duration, clinical outcomes and disease progression

Median time of follow-up after the start of MMF was 51.5 (23.5–115) months in the intolerance group versus 63 (43–120) months in the insufficient response group.

#### 3.5.1 Intolerance group

At the time of diagnosis, 28 patients (72%) had an F0-1, seven (18%) had an F2, and two patients (5%) had an F3-4. In two patients, fibrous collapse was too extensive to grade fibrosis. In one patient information was not available. One patient had severe acute hepatitis with signs of portal hypertension including ascites at presentation but without signs of cirrhosis at diagnosis or during follow-up.

At last follow-up, seven (17.5%) patients were classified as having an advanced fibrosis or cirrhosis, two of those being the ones with extensive fibrous collapse at diagnosis.

#### 3.5.2 Insufficient response group

Four (40%) of the ten patients had F0-1 on initial liver biopsy, three patients (30%) had F2, and two (20%) patients an F4 with evidence of portal hypertension at the time of diagnosis. In one patient, there was no information on fibrosis.

Progression of fibrosis occurred in four (40%) patients, in three patients from F0 to F2 and in one patient from F2 to F3. Of the two patients with cirrhosis at diagnosis, one had a Child score of A6 and another of B8 at last follow-up.

There were no deaths, liver transplantations or listings for liver transplantation in the intolerance or insufficient response group.

Overall, five patients (12.5%) in the intolerance group and five patients (50%) in the insufficient response group had clinical or histological progression of the disease (P = 0.02).

#### 3.5.3 Fibroscan

Median fibroscan value at last follow-up in the intolerance group was 4.9 kPa (4.3–7.1 kPa) and 7.9 kPa (5.9–14.8 kPa) in the insufficient response group (P = 0.184).

### 3.6 Side effects from MMF

Side effects led to discontinuation of MMF in four (8%) patients. MMF was stopped because of agranulocytosis (n = 1), leucopenia (n = 1), hair loss (n = 1) and/or fatigue (n = 2). Three (6%) patients suffered from MMF enteropathy, recurrent topic infections or headaches and symptoms resolved through dose reduction while maintaining remission.

## 4 Discussion

In this study, we report on efficacy and safety of MMF as a second-line treatment in patients with autoimmune hepatitis, delineated by intolerance versus insufficient response to first line-treatment.

Patients switched to second-line treatment with MMF due to intolerance to first-line treatment, achieved disease control on monotherapy in the majority of cases. This high efficacy confirms the value of MMF in the treatment of autoimmune hepatitis in the situation of intolerance to first-line treatment. Previous studies reported complete remission rates on MMF in patients with intolerance to first-line treatment in 43%–92% (Supplementary Table 1) [[15], [16], [17], [18], [19], [20], [21],^26^]. Therefore, the 80% complete remission rate on monotherapy in our cohort is in-line with these previous findings. Comparability, however, is limited, as additional prednisolone dose was reported and accounted for heterogeneously.

Patients switched to MMF due to insufficient response to first-line treatment are less likely to achieve remission on monotherapy with MMF with or without ≤5 mg prednisolone. More commonly, additional prednisolone doses higher than 5 mg/d, switching to third line treatment, and combination treatments were required to control the disease. As monotherapy with MMF achieved remission in 30% of the insufficient response group and led to a reduction in prednisolone doses, it can still be a valuable option in this clinical scenario. Combinations with or switch to third line treatments are important in this population and should be more readily anticipated than in patients with intolerance.

The risk for disease progression was higher in patients who received MMF because of insufficient response. One reason seems to be the more severe disease phenotype. The recommendation to wait six months to evaluate treatment efficacy [2] may need to be individualized in this patient population. Another reason for the higher rate of disease progression might be the difference in the median duration of disease before the start of MMF, which was considerably lower in the intolerance group (six months) in comparison to the insufficient response group (49 months) with longer periods of uncontrolled disease activity.

MMF was overall well tolerated. Intolerance was the reason for discontinuation in 8% among the fifty patients analyzed. This is slightly more than the 6% described in previous publications [14,19], but less than the 34% reported in Hlivko et al. [27]. One patient experienced an agranulocytosis, which was the only severe adverse event and occurred shortly after a COVID-19 infection, which was a relevant additional trigger.

Because of its favorable long-term safety profile, not only monotherapy, but also combination therapy is an important treatment aspect and can help lowering drug levels of treatments with a more problematic side effect profile (such as CI toxicity). This gains further relevance as treatment increasingly focuses on reducing the dosage and duration of steroid exposition [28]. One limitation of MMF is its teratogenic potential, which must be considered in women of childbearing age and men with the wish to father a child [29].

The proportion of patients requiring second line treatment was 44% in our cohort, and therefore higher than the 20% reported in other publications [7,30]. This might be due to referral bias as our cohort is from a tertiary care center.

In contrast to the guidelines published in 2020 [1] for second- and third-line treatment of autoimmune hepatitis, it is not common practice in our center to use 6-mercaptopurine in patients intolerant to AZA. The main reason is the good efficacy and tolerability of MMF in this situation. Furthermore, we have more difficulties obtaining health insurance coverage for 6-mercaptopurine than for MMF.

Our data confirms the importance of MMF as second-line treatment in autoimmune hepatitis, foremost in the situation of intolerance to first-line treatment, but also with value in patients with insufficient response to first-line therapy. Its efficacy as well as its favorable safety profile has led to recent investigations as a first-line treatment in AIH, with superior results in comparison to AZA [31,32], further confirming its importance in the treatment of this rare autoimmune liver disease.

### 4.1 Strengths and limitations of the study

One strength of the study is that the population of patients treated with MMF was the third-largest so far analyzed. The separate analysis on intolerance versus insufficient response as well as the prednisolone doses used are further strengths of the study.

Limitations come from the retrospective character with variation in follow-up periods between patients. Treatment goals and treatment options have changed over the past decades [2], facts that influence disease control, which we could not account or correct for. Only a few patients underwent follow-up liver biopsies, which limits the completeness of the assessment of liver disease progression.

## 5 Conclusions

This study confirms the efficacy and safety of MMF in the treatment of patients with AIH, foremost in the situation of intolerance to first-line treatment. Its effectiveness in patients with insufficient response to first-line treatment is considerably lower and combinations with or switch to third line treatments are important in the population with insufficient response and should be more readily anticipated than in patients with intolerance due to their higher risk of disease progression.

## Funding

MK received financial support from the Swiss 10.13039/501100012451Liver Foundation.

## Authors contributions

KM and DS: conception and design of the study, acquisition of data, analysis and interpretation of data, drafting the article, final approval of the submitted version. DL: analysis of data, revising article critically for important intellectual content, final approval of the submitted version. SGR: acquisition of data, revising article critically for important intellectual content, final approval of the submitted version. BA and SG: revising article critically for important intellectual content, final approval of the submitted version. SN: conception and design of the study, interpretation of data, revising article critically for important intellectual content, final approval of the submitted version

## Declaration of competing interests

The authors declare that they have no known competing financial interests or personal relationships that could have appeared to influence the work reported in this paper.

## Data Availability

Data will be made available on request.

## Appendix A Supplementary data

Multimedia component 1 *Overview on the published literature of MMF in AIH in the adult population.* Response rates are reported separately regarding intolerance and insufficient response if available from the publications Response defined as: Zolfino [33]: International Autoimmune Hepatitis Group report 1993 [34] Hennes [26], Wolf [35], Czaja [36]: reduction in aminotransferase levels to less than twice the normal upper limit (2010 AASLD Guidelines) Devlin [21]: normalization of transaminases Roberts [20]: normalization of transaminases and IgG Chatur [37]: Complete response sustained normalization, partial response improvement by greater than 50%, non-response less than 50% improvement in ALT Hlivko [27]: resolution of symptoms, reduction in transaminases to <2xULN, normalization of serum bilirubin and IgG, improvement in liver histology to normal or only mild portal hepatitis Sharzehi [19]: Refractory disease was defined as persistent elevation of transaminase levels, defined as ≥ 2xULN despite adequate treatment with conventional therapy for at least six months. Partial responders were defined as those with transaminase levels of between 1-2xULN despite treatment for six months. Baven-Pronk [18]: Response: a drop in AST and/or ALT <2xULN 3 months after starting MMF or a 50% fall in AST/or ALT one month after starting MMF. Remission: a normalization of AST and/or ALT after starting MMF. Zachou [14]: complete response when the serum AST and ALT, and gGT or IgG levels had normalized, symptoms had disappeared, and the liver tissue examination, if performed, showed minimal or no inflammation. A partial response was defined as partial decrease of ALT or AST <2xULN without achieving complete normalization and inability to withdraw or taper prednisolone. Non response was defined as persistently elevated transaminase levels more than two times the ULN despite intensive immunosuppression and compliance with therapy. Jothimani [17]: A treatment responder was defined as a patient who developed improvement in their AST and ALT, below twice the upper limit normal 3 months after treatment commencement. Remission was defined as normalization of ALT and or AST after treatment commencement. Efe [15]: Complete biochemical response was defined as normalization of serum aminotransferase and IgG levels at any time within 6 months after starting therapy. Giannakopoulos [16]: Biochemical remission was defined as normalization of serum transaminases. Response was defined as achievement or maintenance of aminotransferase levels ≤ 2xULN. Multimedia component 1

Multimedia component 2 *Real life treatment tree overview of all patients.* n = 141 for remission induction, n = 144 for analysis on first to fourth line treatment. Median follow-up 72 (36–139) months. Other remission induction was AZA in one patient and MMF in one patient. Other first line treatment was prednisolone, etanercept and calcineurin inhibitor in one patient each. Other second line treatment were prednisolone in three patients, azathioprine in one, 6-mercapturine in one and the combination MMF/CI in one patient. Other third line treatment was rituximab in a patient with additionally severe Sjögren's disease. Fourth line treatment was 1) belimumab monotherapy, 2) belimumab/CI/PDN 5 mg, 3) belimumab/CI, 4) CI/Infliximab/PDN 5 mg, 5) MMF/CI/PDN 5 mg, 6) enrolled in a clinical trial PDN, prednisolone; BDN, budesonide; AZA, azathioprine; MMF, mycophenolate mofetil; CI, calcineurin inhibitor Multimedia component 2

Multimedia component 3 *Treatment at last follow-up with separate reporting on use of prednisolone.* n = 144, median follow-up 72 (36–142) months *1) TNFi, PDN 5 mg 2) CI, TNFi, PDN 5 mg 3) MMF, CI, PDN 12.5 mg 4) CI, belimumab 5) CI, belimumab, PDN 5 mg 6) clinical trial Of those in remission without treatment, 16 patients (59%) had been on AZA, 8 (30%) on MMF, one on budesonide, one on prednisolone and one on belimumab monotherapy. PDN, prednisolone; AZA, azathioprine; MMF, mycophenolate mofetil; CI, calcineurin inhibitor; TNFi, tumor necrosis factor inhibitor Multimedia component 3

Multimedia component 4 *Data on remission of all patients at last follow-up.* n = 144 for ALT, n = 135 for IgG and ALT/IgG, median follow-up 72 (36–142) months *50% of patients with IgG >1.1xULN had a cirrhosis or a connective tissue disease as an additional reason for the elevation. ALT, alanine aminotransferase; IgG, Immunoglobulin G Multimedia component 4

## References

[1] Lohse A.W., Sebode M., Jorgensen M.H., Ytting H., Karlsen T.H., Kelly D. (2020). Second-line and third-line therapy for autoimmune hepatitis: a position statement from the European reference network on hepatological diseases and the international autoimmune hepatitis group. J. Hepatol..

[2] Pape S., Snijders R.J., Gevers T.J., Chazouilleres O., Dalekos G.N., Hirschfield G.M. (2022). Systematic review of response criteria and endpoints in autoimmune hepatitis by the International Autoimmune Hepatitis Group. J. Hepatol..

[3] Soloway R.D., Summerskill W.H., Baggenstoss A.H., Geall M.G., Gitnick G.L., Elveback I.R. (1972). Clinical, biochemical, and histological remission of severe chronic active liver disease: a controlled study of treatments and early prognosis. Gastroenterology.

[4] Cook G.C., Mulligan R., Sherlock S. (1971). Controlled prospective trial of corticosteroid therapy in active chronic hepatitis. Q. J. Med..

[5] Murray-Lyon I.M., Stern R.B., Williams R. (1973). Controlled trial of prednisone and azathioprine in active chronic hepatitis. Lancet.

[6] Summerskill W.H., Korman M.G., Ammon H.V., Baggenstoss A.H. (1975). Prednisone for chronic active liver disease: dose titration, standard dose, and combination with azathioprine compared. Gut.

[7] Lamers M.M., van Oijen M.G., Pronk M., Drenth J.P. (2010). Treatment options for autoimmune hepatitis: a systematic review of randomized controlled trials. J. Hepatol..

[8] Czaja A.J. (2009). Current and future treatments of autoimmune hepatitis. Expet Rev. Gastroenterol. Hepatol..

[9] Seela S., Sheela H., Boyer J.L. (2005). Autoimmune hepatitis type 1: safety and efficacy of prolonged medical therapy. Liver Int..

[10] Wiesner R., Rabkin J., Klintmalm G., McDiarmid S., Langnas A., Punch J. (2001). A randomized double-blind comparative study of mycophenolate mofetil and azathioprine in combination with cyclosporine and corticosteroids in primary liver transplant recipients. Liver Transplant..

[11] Allison A.C. (2005). Mechanisms of action of mycophenolate mofetil. Lupus.

[12] Vollmar Angelika D.T. (2005).

[13] Schuppan D.H.C., Strobel D. (1998). Successful treatment of therapy-refractory autoimmune hepatitis with mycophenolate mofetil. Hepatology.

[14] Zachou K., Gatselis N., Papadamou G., Rigopoulou E.I., Dalekos G.N. (2011). Mycophenolate for the treatment of autoimmune hepatitis: prospective assessment of its efficacy and safety for induction and maintenance of remission in a large cohort of treatment-naive patients. J. Hepatol..

[15] Efe C., Hagstrom H., Ytting H., Bhanji R.A., Muller N.F., Wang Q. (2017). Efficacy and safety of mycophenolate mofetil and tacrolimus as second-line therapy for patients with autoimmune hepatitis. Clin. Gastroenterol. Hepatol..

[16] Giannakopoulos G., Verbaan H., Friis-Liby I.L., Sangfelt P., Nyhlin N., Almer S. (2019). Mycophenolate mofetil treatment in patients with autoimmune hepatitis failing standard therapy with prednisolone and azathioprine. Dig. Liver Dis..

[17] Jothimani D., Cramp M.E., Cross T.J. (2014). Role of mycophenolate mofetil for the treatment of autoimmune hepatitis-an observational study. J Clin Exp Hepatol.

[18] Baven-Pronk A.M., Coenraad M.J., van Buuren H.R., de Man R.A., van Erpecum K.J., Lamers M.M. (2011). The role of mycophenolate mofetil in the management of autoimmune hepatitis and overlap syndromes. Aliment. Pharmacol. Ther..

[19] Sharzehi K., Huang M.A., Schreibman I.R., Brown K.A. (2010). Mycophenolate mofetil for the treatment of autoimmune hepatitis in patients refractory or intolerant to conventional therapy. Can. J. Gastroenterol..

[20] Roberts S.K., Lim R., Strasser S., Nicoll A., Gazzola A., Mitchell J. (2018). Efficacy and safety of mycophenolate mofetil in patients with autoimmune hepatitis and suboptimal outcomes after standard therapy. Clin. Gastroenterol. Hepatol..

[21] Devlin S.M., Swain M.G., Urbanski S.J., Burak K.W. (2004). Mycophenolate mofetil for the treatment of autoimmune hepatitis in patients refractory to standard therapy. Can. J. Gastroenterol..

[22] Liberal R., de Boer Y.S., Andrade R.J., Bouma G., Dalekos G.N., Floreani A. (2017). Expert clinical management of autoimmune hepatitis in the real world. Aliment. Pharmacol. Ther..

[23] Hennes E.M., Zeniya M., Czaja A.J., Pares A., Dalekos G.N., Krawitt E.L. (2008). Simplified criteria for the diagnosis of autoimmune hepatitis. Hepatology.

[24] Bedossa P., Poynard T. (1996). An algorithm for the grading of activity in chronic hepatitis C. The METAVIR Cooperative Study Group. Hepatology.

[25] (2015). European association for the study of the L. EASL clinical practice guidelines: autoimmune hepatitis. J. Hepatol..

[26] Hennes E.M., Oo Y.H., Schramm C., Denzer U., Buggisch P., Wiegard C. (2008). Mycophenolate mofetil as second line therapy in autoimmune hepatitis?. Am. J. Gastroenterol..

[27] Hlivko J.T., Shiffman M.L., Stravitz R.T., Luketic V.A., Sanyal A.J., Fuchs M. (2008). A single center review of the use of mycophenolate mofetil in the treatment of autoimmune hepatitis. Clin. Gastroenterol. Hepatol..

[28] van den Brand F.F., van der Veen K.S., Lissenberg-Witte B.I., de Boer Y.S., van Hoek B., Drenth J.P.H. (2019). Adverse events related to low dose corticosteroids in autoimmune hepatitis. Aliment. Pharmacol. Ther..

[29] Sammaritano L.R., Bermas B.L., Chakravarty E.E., Chambers C., Clowse M.E.B., Lockshin M.D. (2020). American college of rheumatology guideline for the management of reproductive health in rheumatic and musculoskeletal diseases. Arthritis Care Res..

[30] Czaja A.J. (2009). Mycophenolate mofetil to the rescue in autoimmune hepatitis: a fresh sprout on the decision tree. J. Hepatol..

[31] Dalekos G.N., Arvaniti P., Gatselis N.K., Samakidou A., Gabeta S., Rigopoulou E. (2021). First results from a propensity matching trial of mycophenolate mofetil vs. Azathioprine in treatment-naive AIH patients. Front. Immunol..

[32] Dalekos Gn A.P., Gatselis N.K., Gabeta S., Samakidou A., Giannoulis G., Rigopoulou E., Koukoulis G.K., Zachou K. (2022). Long-term results of mycophenolate mofetil vs. azathioprine use in patients with autoimmune hepatitis. J HEP reports.

[33] Zolfino T., Heneghan M.A., Norris S., Harrison P.M., Portmann B.C., McFarlane I.G. (2002). Characteristics of autoimmune hepatitis in patients who are not of European Caucasoid ethnic origin. Gut.

[34] Johnson P.J., McFarlane I.G. (1993). Meeting report: international autoimmune hepatitis group. Hepatology.

[35] Wolf D.C., Bojito L., Facciuto M., Lebovics E. (2009). Mycophenolate mofetil for autoimmune hepatitis: a single practice experience. Dig. Dis. Sci..

[36] Czaja A.J., Carpenter H.A. (2005). Empiric therapy of autoimmune hepatitis with mycophenolate mofetil: comparison with conventional treatment for refractory disease. J. Clin. Gastroenterol..

[37] Chatur N., Ramji A., Bain V.G., Ma M.M., Marotta P.J., Ghent C.N. (2005). Transplant immunosuppressive agents in non-transplant chronic autoimmune hepatitis: the Canadian association for the study of liver (CASL) experience with mycophenolate mofetil and tacrolimus. Liver Int..

