# Supplementary material for: Mycophenolate mofetil as second line treatment in autoimmune hepatitis – A retrospective single center analysis
Source: J Transl Autoimmun. 2022 Nov 19;5:100172. doi: 10.1016/j.jtauto.2022.100172 (PMC9702977; doi:10.1016/j.jtauto.2022.100172)
Supplement: Multimedia component 1 — Overview on the published literature of MMF in AIH in the adult population. Response rates are reported separately regarding intolerance and insufficient response if available from the publications Response defined as: Zolfino [33]: International Autoimmune Hepatitis Group report 1993 [34] Hennes [26], Wolf [35], Czaja [36]: reduction in aminotransferase levels to less than twice the normal upper limit (2010 AASLD Guidelines) Devlin [21]: normalization of transaminases Roberts [20]: normalization of transaminases and IgG Chatur [37]: Complete response sustained normalization, partial response improvement by greater than 50%, non-response less than 50% improvement in ALT Hlivko [27]: resolution of symptoms, reduction in transaminases to <2xULN, normalization of serum bilirubin and IgG, improvement in liver histology to normal or only mild portal hepatitis Sharzehi [19]: Refractory disease was defined as persistent elevation of transaminase levels, defined as ≥ 2xULN despite adequate treatment with conventional therapy for at least six months. Partial responders were defined as those with transaminase levels of between 1-2xULN despite treatment for six months. Baven-Pronk [18]: Response: a drop in AST and/or ALT <2xULN 3 months after starting MMF or a 50% fall in AST/or ALT one month after starting MMF. Remission: a normalization of AST and/or ALT after starting MMF. Zachou [14]: complete response when the serum AST and ALT, and gGT or IgG levels had normalized, symptoms had disappeared, and the liver tissue examination, if performed, showed minimal or no inflammation. A partial response was defined as partial decrease of ALT or AST <2xULN without achieving complete normalization and inability to withdraw or taper prednisolone. Non response was defined as persistently elevated transaminase levels more than two times the ULN despite intensive immunosuppression and compliance with therapy. Jothimani [17]: A treatment responder was defined as a patient who developed impr [file mmc1.docx]

| **Study** | **Year of publication** | **Number of patients on MMF** | **Follow-up under MMF treatment in months** | **Response to MMF overall (%)** | **Complete response if intolerance was the cause (%)** | **Complete response if insufficient response was the cause (%)** | **Of note** |
| --- | --- | --- | --- | --- | --- | --- | --- |
| Richardson et al | 2000 | 7 | Median 46 (21-59) | 6/7 (85.7%) complete response without prednisolone  1/7 (14.3%) complete response with 8mg prednisolone |  |  |  |
| Zolfino et al | 2002 | 2 | Not reported | 0/2 (0%) insufficient response |  |  | MMF part of a multidrug regiment to control highly difficult to treat disease |
| Devlin et al | 2004 | 5 | Mean 19.8 (12-36) | 2/5 (40%) without prednisolone  3/5 (60%) with 5-10mg of prednisolone | 2/4 (50%) on MMF monotherapy  2/4 (50%) with 5-10mg prednisolone | 1/1 (100%) with ongoing 5mg prednisolone |  |
| Chatur et al | 2005 | 11 (plus 2 on MMF and Tacrolimus, not included in this table) | Mean 26.5 (10-54) | 7/11 (64%) complete response  2/11 (18%) relapsed after complete response  1/11 (9%) partial response  1/11 (9%) non response (died while waiting for liver transplant) |  |  | The mean pretreatment prednisolone dose was 20mg and the mean post treatment prednisolone dose was 4.7mg. Prednisolone dose was also markedly reduced in the partial responders.  Four patients could stop prednisolone. |
| Czaja et al | 2005 | 8 | Mean 19 (4-60) | 0/8 (0%) complete response  5/8 (62%) improvement or stable suppression of ASAT activity  3/8 (38%) non response |  | 0/7 (0%) complete response | None of the patients had responded satisfactorily to prior prednisolone treatment.  MMF did not have a sufficient effect in the patients that failed standard treatment with prednisolone. |
| Inductivo-Yu et al | 2007 | 15 | Mean 41 | 4/15 (27%) complete response on MMF monotherapy  6/15 (40%) complete response on MMF and prednisolone (5-10mg/d)  5/15 (33%) stopped MMF (3 were switched to tacrolimus,  1 stopped due to non-compliance,  1 loss of follow up) |  |  | 11/15 (73%) where switched to MMF because of insufficient response  4/15 (27%) because of intolerance |
| Hlivko et al | 2008 | 29 | Not reported | 16/29 (55%) overall remission including patients not tolerating MMF  16/19 (84%) first and second-line on MMF together in patients that tolerated MMF  8/12 (67%) CR second line on MMF (9 due to intolerance, 3 due to insufficient response), most still on additional prednisolone |  |  | 17 patients with MMF as first line treatment  Intolerance to MMF in 34% |
| Hennes et al | 2008 | 36 |  | 14/36 (39%) complete response  22/36 (61%) incomplete response | 12/28 (43%) | 2/8 (25%) |  |
| Wolf et al | 2009 | 16 | Median 23 (1.4-94.9) | 12/16 (75%) good response  8/16 (50%) normalization of ALT  Median prednisolone dose decreased from 10mg/d to 3mg/d |  |  | 7/16 (44%) switched to MMF due to intolerance to AZA, 6/16 (37.5%) due to insufficient response, 3/16 (18.5%) other reasons  1/17 (6%) intolerance to MMF |
| Sharzehi et al | 2010 | 17 |  | 8/21 (38%)  Mean decrease in steroids from 18.9mg/d to 7.8mg/d | 8/9 (88%) | 0/12 (0%)  all showed biochemical improvement, but no complete response | 1/17 (5.8%) with intolerance to MMF |
| Baven-Pronk et al | 2011 | 45 | Median 39.5 (3-133) |  | 10/15 (67%) complete response  0/15 (0%) partial response  2/8 (25%) with overlap remission  5/8 (63%) with overlap response | 2/15 (13%) remission  4/15 (27%) response  4/7 (57%) with overlap remission  1/7 (14%) with overlap response | 33% had side effects, 13% discontinued MMF  In the AZA-intolerance group 20% had a cirrhosis before MMF, in the AZA-non-responders 73%  2 on MMF monotherapy in remission. Mean prednisolone dose of 10mg in all patients |
| Zachou et al | 2011 | 59 | Median follow-up 42 (3-117) | 35/59 (59.3%) with complete response: 22/59 (37%) CR off steroids, 13/59 (22.3%) CR but still in the steroid tapering period  17/59 (28.8%) initial CR with steroid dependency  6/59 (10.2%) PR  1/59 (1.7%) CR but only partial improvement on histology |  |  | Prospective, MMF used as first-line treatment  4/59 (6.8%) adverse events considered to MMF with discontinuation (2 septicemia in cirrhotic patients) or dose reduction due to leucopenia in 2 patients with advanced fibrosis |
| Jothimani et al | 2014 | 19 | Median 47 (5-83) | 14/20 (73.6%) biochemical remission  Thereof 4/5 (80%) patients with cirrhosis in biochemical remission | 14/18 (78%) biochemical remission | 1/2 (50%) initial response with relaps  1/2 (50%) without response | 3/20 intolerant to MMF (rash, hair loss)  1 lost follow up  8/14 (57%) maintained prednisolone at lower dose, 4/14 (28.5%) MMF monotherapy |
| Park et al | 2016 | 1 |  |  | 1/1 (100%) MMF with prednisolone 5 mg |  |  |
| Efe et al | 2017 | 121 | Median 62 (6-190) | 84/121 (69.4%) | 68/74 (91.9%) | 16/47 (34%) | 26/121 (21.5%) prednisolone could be stopped  10/121 (13.2%) withdrawal of MMF due to side effects  7 non-responders to MMF showed a complete response to tacrolimus  6/8 (75%) of patients on MMF and tacrolimus showed complete remission |
| Roberts et al | 2018 | 105 | Median 34 (18-69) | 63/105 (60%) complete response  34/105 (32%) incomplete response  8/105 (8%) treatment failure  Non-cirrhotic patients: 42/64 (65.6%) complete remission  Cirrhotic patients: 18/38 (47.3%) complete remission | 39/63 (62%) complete response  20/63 (32%) incomplete response  4/63 (6.3%) treatment failure  Cirrhotic patients 18/38 (50%) complete remission | 24/42 (57%) complete response  14/42 (33%) incomplete response  4/42 (9.5%) treatment failure  Cirrhotic patients 18/38 (44%) complete remission | A lower proportion of patients with cirrhosis achieved biochemical remission (47% versus 60%), with no difference regarding treatment indication.  3/105 (2.9%) serious adverse events (including one death). 10/105 (9.5%) discontinued MMF due to side effects.  No information about prednisolone dosage. |
| Giannakopoulos et al | 2019 | 22 | Median 71 (20-124) | 10/22 (45%) normal transaminases  4/6 (60%) patients with cirrhosis normalized transaminases, but 4/6 stopped MMF due to side effects | 9/14 (64%) | 1/5 (20%) | Overall prednisolone was reduced from 19 mg/d to 4.5mg/d  12/22 (55%) stopped MMF after 1-6 months,6 due to adverse events (gastrointestinal, rash, headache), 4 with lack of biochemical control, 1 with recurrence of lymphoma and 1 with the wish to conceive  6/20 (30%) cirrhotic with more adverse events |
