# Supplementary figures and images for: Mycophenolate mofetil as second line treatment in autoimmune hepatitis – A retrospective single center analysis
Source: J Transl Autoimmun. 2022 Nov 19;5:100172. doi: 10.1016/j.jtauto.2022.100172 (PMC9702977; doi:10.1016/j.jtauto.2022.100172)

## Real life treatment tree overview of all patients

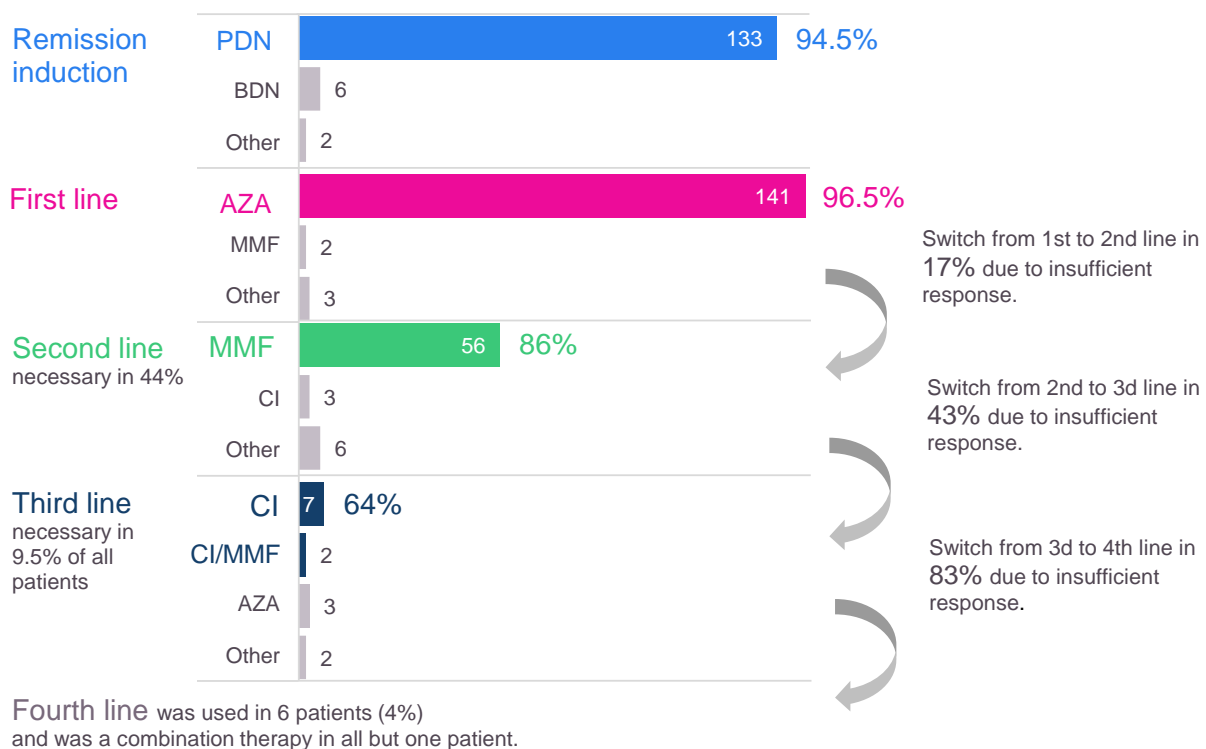

Supplement: Multimedia component 2 — Real life treatment tree overview of all patients. n = 141 for remission induction, n = 144 for analysis on first to fourth line treatment. Median follow-up 72 (36–139) months. Other remission induction was AZA in one patient and MMF in one patient. Other first line treatment was prednisolone, etanercept and calcineurin inhibitor in one patient each. Other second line treatment were prednisolone in three patients, azathioprine in one, 6-mercapturine in one and the combination MMF/CI in one patient. Other third line treatment was rituximab in a patient with additionally severe Sjögren's disease. Fourth line treatment was 1) belimumab monotherapy, 2) belimumab/CI/PDN 5 mg, 3) belimumab/CI, 4) CI/Infliximab/PDN 5 mg, 5) MMF/CI/PDN 5 mg, 6) enrolled in a clinical trial PDN, prednisolone; BDN, budesonide; AZA, azathioprine; MMF, mycophenolate mofetil; CI, calcineurin inhibitor [file mmc2.pdf]

## Treatment overview of all patients at last follow-up

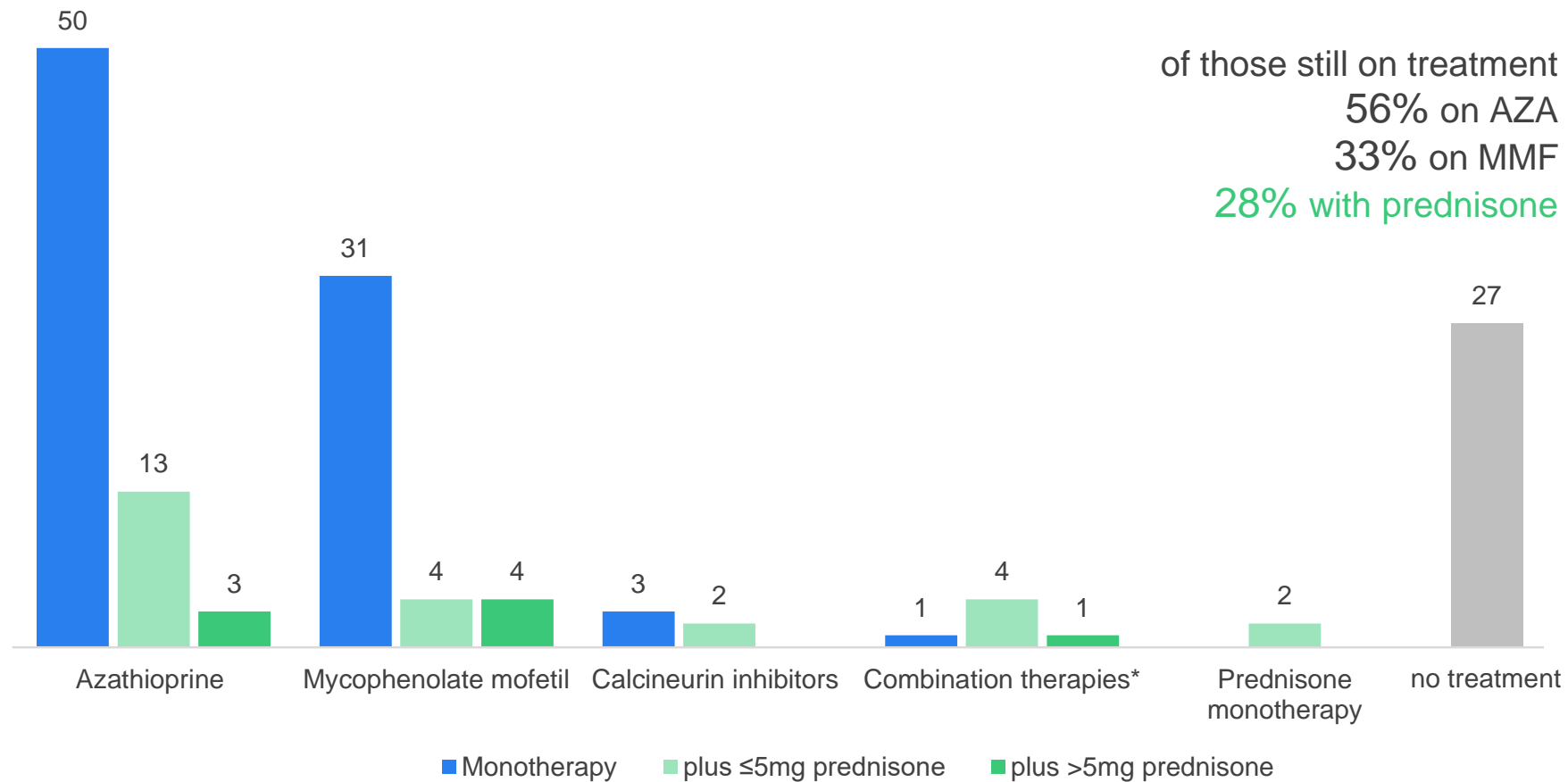

Supplement: Multimedia component 3 — Treatment at last follow-up with separate reporting on use of prednisolone. n = 144, median follow-up 72 (36–142) months *1) TNFi, PDN 5 mg 2) CI, TNFi, PDN 5 mg 3) MMF, CI, PDN 12.5 mg 4) CI, belimumab 5) CI, belimumab, PDN 5 mg 6) clinical trial Of those in remission without treatment, 16 patients (59%) had been on AZA, 8 (30%) on MMF, one on budesonide, one on prednisolone and one on belimumab monotherapy. PDN, prednisolone; AZA, azathioprine; MMF, mycophenolate mofetil; CI, calcineurin inhibitor; TNFi, tumor necrosis factor inhibitor [file mmc3.pdf]

## Proportions of remission of all patients at last follow-up

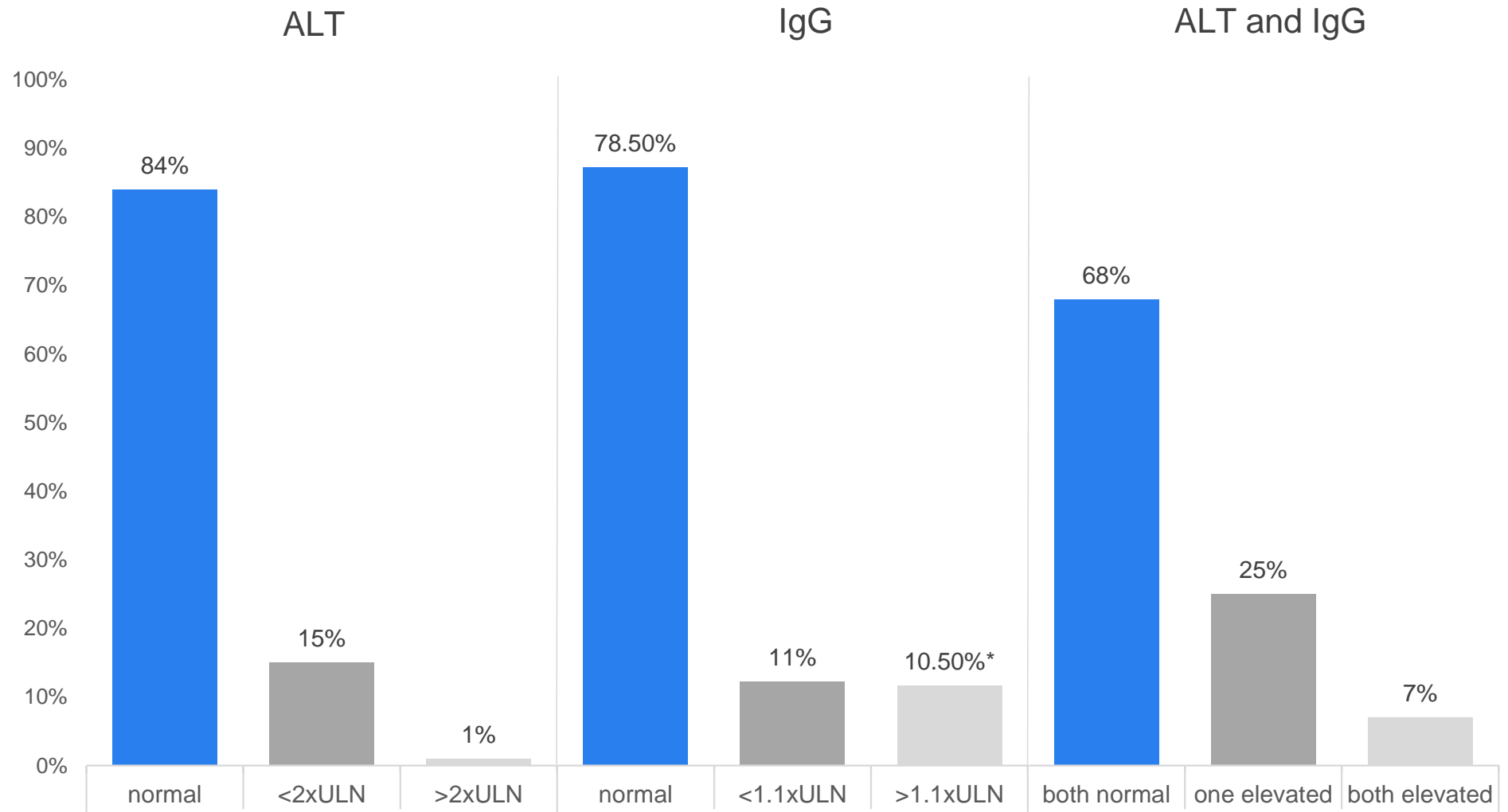

Supplement: Multimedia component 4 — Data on remission of all patients at last follow-up. n = 144 for ALT, n = 135 for IgG and ALT/IgG, median follow-up 72 (36–142) months *50% of patients with IgG >1.1xULN had a cirrhosis or a connective tissue disease as an additional reason for the elevation. ALT, alanine aminotransferase; IgG, Immunoglobulin G [file mmc4.pdf]
